# Spatio-temporal pattern evolution and dynamic simulation of urban ecological resilience in Guangdong Province, China

**DOI:** 10.1016/j.heliyon.2024.e25127

**Published:** 2024-01-26

**Authors:** Zhenjie Liao, Lijuan Zhang

**Affiliations:** School of Management, Guangzhou Huashang College, Guangzhou, China

**Keywords:** Urban ecological resilience, Dynamic evolution, Obstacle factors, Back propagation neural network model, Guangdong province

## Abstract

Currently, in-depth analyses concerning the dynamic simulation of urban resilience and forecasting future development trends are lacking. To address urban vulnerability and promote regional balance and sustainable development, this study assessed the urban ecological resilience of Guangdong Province from 2000 to 2020 using the entropy weight TOPSIS method. Furthermore, we examined the spatial and temporal variations and evolution of urban ecological resilience through measures such as kernel density estimation, Theil index, and the center of gravity standard deviation ellipse. We employ obstacle degree and back-propagation (BP) neural network models to identify the primary barriers and conduct dynamic simulations. Our findings revealed that, from an evolutionary resilience perspective, urban ecological resilience is an inherent characteristic of urban ecosystems. It consistently possesses the dynamic ability to defend against disturbances, respond promptly when interference occurs, and continually learn and innovate, regardless of the urban ecology's state of disturbance. Urban ecological resilience in Guangdong Province has steadily improved with minimal fluctuations, establishing a trend characterized by low concentration and high convergence. Regarding barrier factors, the disposal rate of domestic waste, number of college students per 10,000 people, number of R&D personnel per 10,000 labor force, and per capita park green space area are the primary constraints on urban ecological resilience in Guangdong Province. Dynamic simulations from 2022 to 2030 suggest that urban resilience will experience gradual development with a decreasing overall resilience level. Areas with lower and median resilience values will predominate, while the number of cities with higher resilience levels will see a reduction. Future development trends indicate notable temporal and spatial variations. In the east and west directions, the urban resilience level forms a “U” shape, while in the north and south directions, it is higher in the south and lower in the north.

## 1 Introduction

Urban agglomerations, as complex city systems, face various challenges, including natural disasters and human-induced pressures resulting from the continuous concentration of population and industries within cities [1]. These disturbances are often unpredictable and hard to mitigate. Therefore, enhancing the level of urban resilience in urban agglomerations to deal with these disruptions, boost regional resilience, and promote sustainable urban development has become a prominent topic in 21st-century urban geography and urban planning [2]. Understanding and assessing the current resilience of cities in Guangdong Province can serve as a foundation and practical guide for its enhancement.

The term “resilience” originates from the Latin word “Resilio,” which means “return to the original state” [3]. Over time, the concept of resilience has undergone significant transformations, progressing from engineering resilience to ecological resilience and, ultimately, to evolutionary resilience. Engineering resilience primarily focuses on a system's capacity to return to its initial equilibrium state after experiencing disturbances [4]. However, as our understanding of system characteristics and mechanisms deepened within the academic community, the traditional engineering resilience paradigm began to appear inflexible and one-dimensional. In 1973, Holling extended the concept of resilience to the realm of natural ecology, introducing the concept of ecological resilience [5]. Ecological resilience emphasizes a system's ability to recover to its original state or establish a new equilibrium state in the face of disturbances [6]. As systems became increasingly complex, the ecological resilience paradigm also encountered challenges in terms of its explanatory power, giving rise to the concept of evolutionary resilience.

Evolutionary resilience shifts the focus from returning to a balanced state to a system's ability to achieve sustainable development by adjusting its structure and altering its trajectory [7]. In summary, two dominant perspectives on resilience understanding exist equilibrium and evolution theories. Both engineering and ecological resilience fall under the equilibrium theory perspective, emphasizing a system's recovery capacity following interference [8]. Evolutionary resilience, rooted in the evolution theory perspective, posits that resilience is an inherent system attribute independent of external disruptions and encompasses three vital characteristics: defense, response, and adaptability [9]. Among these perspectives, the view of evolutionary resilience holds greater theoretical persuasiveness and should serve as a reference for urban resilience research. However, it is essential to acknowledge and engage in discussions regarding the evolving nature of the resilience concept itself. Recent resilience approaches generally align with the non-equilibrium resilience model [10].

A comprehensive review and analysis of existing literature revealed that scholars have primarily focused on several key aspects of urban resilience.

(1) Urban resilience and climate change: Research in this area addresses climate change challenges and urban adaptability, including topics [11] such as, the connection between tourism and urban ecological resilience [12], and strategies involving structural and non-structural measures to enhance community resilience in flood-prone areas [13].
(2) Urban resilience and urban planning/construction: Scholars have explored how urban planning and construction can contribute to resilience. This includes integrating cities with their natural environments, implementing urban planning approaches like water and mountain-friendly building designs, green buildings, and flood adaptation models aimed at enhancing urban development resilience [14,15].
(3) Urban resilience governance models: Researchers have employed semi-structured interviews to investigate the interactions among key elements of urban resilience. This has led to the development of adaptive governance frameworks and decentralized multi-center governance models [16,17].
(4) Social dynamic mechanism of urban resilience: Cowell and Hudec analyzed how factors like urban economic development and unemployment growth rates impact urban resilience and proposed the resilience index (RCI) [18,19].
(5) Urban resilience and landscape ecology: McClintock explored the sustainable development of American residential garden cities, considering social spatial differentiation [20,21].

In recent years, amid crises driven by globalization, urbanization, and ecological vulnerabilities, urban ecological resilience has gained significant attention from both academia and society as a critical dimension of urban resilience assessment [22]. However, research on this topic remains relatively new and contentious. Current literature primarily centers on urban planning and risk mitigation.

In urban planning, researchers have analyzed ecological resilience at the urban level, constructed indicator systems for urban ecological resilience, and discussed urban development pathways aligned with new urbanization [23]. Others have integrated ecological resilience with urban governance performance to develop planning systems for complex urban systems [24]. Some scholars have assessed urban water ecological resilience and explored urban development approaches guided by water environments through the lens of ecological resilience [25].

Regarding urban risk mitigation, studies have constructed assessment models for urban ecological resilience and analyzed spatio-temporal patterns of change [26]. At the micro-level, researchers have analyzed the resilience of urban communities to disasters and provided targeted recommendations [27]. Urban ecological resilience measurement has been undertaken for various cities in Yunnan Province, focusing on ecosystem “defense capability,” “response capability,” and “learning capability” [28]. Additionally, Li introduced resilience concepts into the human settlement environment, leading to the development of evaluation index systems for urban human settlement resilience in regions like the Yangtze River Delta [29].

Notably, research on urban ecological resilience has primarily focused on analyzing the “level” of urban ecosystems in terms of their resistance to interference, restoration, and construction due to limited data availability and the evolving definition of the concept. There is a relative scarcity of discussions on urban ecological resilience from the perspective of evolutionary resilience, which makes it challenging to comprehensively assess the core essence and innovative development capacities of urban ecosystems.

A comprehensive examination of current research progress in urban resilience reveals a broad spectrum of research content. Existing studies primarily revolve around the establishment and planning of resilient cities [30], the quantification of urban resilience [31], the investigation into the spatial differentiation mechanisms affecting urban resilience [32], and the optimization of development strategies [33]. However, there is a noticeable gap in conducting in-depth analyses concerning the dynamic simulation of urban resilience and forecasting future development trends. In terms of research methodologies, the predominant approaches involve traditional mathematical and physical characteristic analyses, along with index evaluations and GIS spatial analyses. The integrated application of multiple methods and approaches is relatively uncommon. In relation to research scope, the majority of studies focus on the provincial and municipal levels. Nevertheless, considering the influence of interconnections and nesting across various spatial scales, exploring urban resilience from medium and macro perspectives remains essential.

Therefore, adopting the viewpoint of evolutionary resilience, this study conceived urban ecological resilience as an inherent attribute of urban ecosystems. Irrespective of whether urban ecology faces disturbance, it consistently possesses dynamic capabilities: preemptive defense against interference, timely responses when disturbances occur, and a continuous learning and innovation process. Building upon this foundation, the study draws inspiration from the dynamic evolution characteristics of the adaptive cycle model, delineating three stages of urban ecological resilience: resistance, response, and renewal. The resistance capacity of an urban ecosystem is characterized by its ability to withstand disturbances while maintaining function and structure unchanged. Responsiveness signifies the aptitude to swiftly and diversely counter impacts during disturbances. Renewal capability manifests as heightened responsiveness to interference, leading to breakthroughs in pathways and structural renewal within urban ecosystems through learning and innovation.

Based on the aforementioned analysis, Guangdong Province serves as a representative case study. Utilizing panel data spanning from 2000 to 2020, we construct an assessment index system for urban ecological resilience, emphasizing the dimensions of “resistance, response, and renewal.” We employed the entropy weight TOPSIS method to comprehensively evaluate the urban ecological resilience of Guangdong Province. Additionally, leveraging tools such as kernel density estimation, the Theil index, and the gravity center standard deviation ellipse, we delved into the spatio-temporal pattern evolution of urban ecological resilience within the province. Finally, we analyzed the hindering factors and assess the level of urban ecological resilience in Guangdong Province through an obstacle degree model. We anticipate that our findings will offer theoretical underpinnings and a quantitative foundation to support urban ecological preservation and the development of high-quality cities in Guangdong Province.

## 2 Data and methods

### 2.1 Study area

**Fig. 1:**
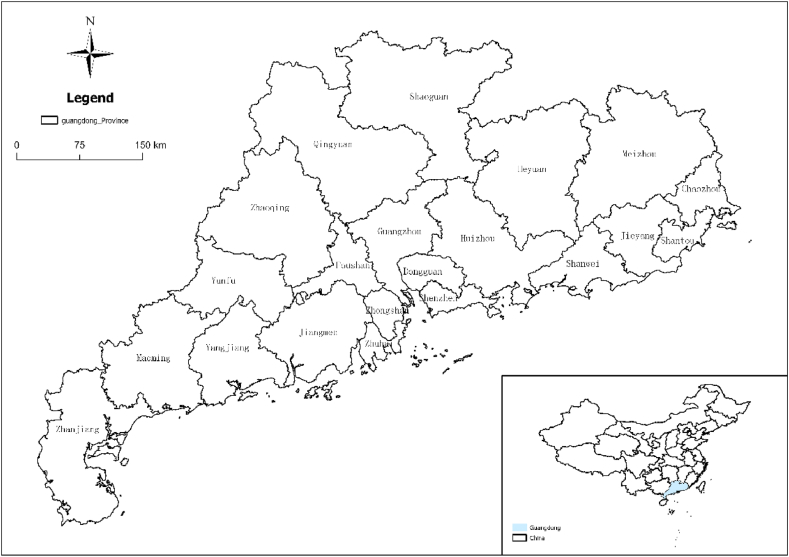
Study area overview.

Guangdong Province is located in southern China, bordering Fujian to the east, Jiangxi and Hunan to the north, Guangxi to the west, and the South China Sea to the south, between 20°09′ N–25°31′N and 109°45′E−117°20′E (Fig. 1). As the province boasting the largest economy and population in China, Guangdong Province recorded a remarkable GDP of 12.43 trillion RMB and a stable resident population of 126 million in the year 2020. Its favorable economic and social development conditions continually attract an influx of population and resources from other provinces, establishing Guangdong Province as a focal point for the movement of people and goods across the nation. However, The rapid population growth and extensive urbanization have imposed substantial pressures on resources and the environment. This has consequently given rise to a multitude of ecological and environmental challenges, including the heightened frequency of flood disasters, the reduction of wetland areas, and the decline in both biodiversity and water conservation capacity.

### 2.2 Indicator system and data

#### 2.2.1 Index system construction

Drawing upon relevant literature and existing research findings and guided by the principles of urban resilience theory and sustainable development theory, this study aims to establish a comprehensive indicator system for assessing urban ecological resilience. The assessment focuses on three fundamental dimensions: resistance, response, and renewal, providing an objective evaluation of Guangdong Province's urban ecological resilience. The specific indicators considered in each dimension are as follows.

(1) Resistance dimension: resistance is contingent upon the initial conditions inherent in the system, such as the ecological background. Building upon prior scholarly work, we have curated ecological factors encompassing water, soil, and biological resources [34,35]. The selected indicators in this dimension encompass per capita water resources [36], per capita park area, normalized vegetation index, and biological abundance index [37].
(2) Response dimension: Recognizing that urban ecosystems possess social attributes, the capacity to respond underscores the measures taken by social agents in response to disturbances. Accordingly, we have identified indicators that capture these social responses, including the production of general industrial solid waste, the centralized treatment rate of sewage treatment plants, the harmless treatment rate of domestic waste, and the number of environmental protection monitoring stations.
(3) Renewal dimension: In line with the concept of evolutionary resilience, where resilient evolution primarily hinges on “innovation intensity adaptation” [38], the renewal dimension underscores innovation intensity. This dimension manifests as indicators related to innovation output, innovation input, and innovation intensity [39,40]. Therefore, we have selected indicators such as R&D investment [41], the number of patent licenses, the number of R&D personnel, and the number of college students [42]. The renewal dimension serves as a valuable complement to the assessment of ecological resilience, addressing the specific context and representing an innovative addition to the construction of the urban ecological resilience evaluation system outlined in this article.

To determine the appropriate weights for these indicators, we employ a comprehensive weighting methodology that combines the entropy method with the Analytic Hierarchy Process, yielding a reliable weighting scheme (see Table 1).

**Table 1:** Evaluation index system of urban ecological resilience in Guangdong province.

Target layer	One grade indexes	Two grade indexes	Weight	Number
Urban ecological resilience	Resistance dimension	Per capita water resources [36]	0.0695	RS1
		Per capita park area	0.0984	RS2
		Normalized vegetation index	0.0175	RS3
		Biological abundance index [37]	0.0917	RS4
		The production of general industrial solid waste	0.0129	RP5
	Response dimension	Centralized treatment rate of sewage treatment plants	0.0061	RP6
		Harmless treatment rate of domestic waste	0.1278	RP7
		Number of environmental protection monitoring stations	0.0942	RP8
	Renewal dimension	R&D investment [41]	0.0244	IN9
		Number of patent licenses	0.1729	IN10
		Number of R&D personnel	0.101	IN11
		Number of college students [42]	0.1836	IN12

#### 2.2.2 Data source

The data utilized in this research primarily originated from the China Urban Statistical Yearbook (2001–2021) and the Guangdong Statistical Yearbook (2001–2021). Some of the processed data, including the biological abundance index, per capita water resources, and per capita park green space area, were derived through calculations involving various indicator combinations. Furthermore, the NDVI data for the 21 prefecture-level cities within Guangdong Province, featuring a 30-m resolution, were procured from the Resource and Environmental Science Data Center of the Chinese Academy of Sciences (http://www. resdc. cn/).

### 2.3 Research methods

#### 2.3.1 Entropy weight TOPSIS

We referred to the research methods of Chen [43] and others to evaluate the urban ecological resilience of Guangdong Province using the entropy weight TOPSIS method. In contrast to the traditional TOPSIS evaluation method, the entropy weight TOPSIS method has the advantages of intuitive geometric meaning, reduced information loss, and flexible operation. The specific steps are as follows [44]:

(1) $$e_{j}=-k\sum_{i=1}^{m}\left[\left(y_{ij}/\sum_{i=1}^{m}y_{ij}\right)\cdot \ln\left(y_{ij}/\sum_{i=1}^{m}y_{ij}\right)\right]，$$

k=1/lnm

(2) $$w_{j}=\left(1-e_{j}\right)/\sum_{j=1}^{12}\left(1-e_{j}\right)$$

where *w*_*j*_ is the weight of index *j*; *e*_*j*_ represents the information entropy of index *j*; *m* is the number of evaluation years; *k* is the Boltzmann constant.

② Determine the positive ideal solution and negative ideal solution, and calculate the Euclidean distance [45]:

(3) $$\text{Positive}\;\text{ideal}\;\text{solution}\;S_{i}^{+}=\sqrt{\sum_{j=1}^{m}{\left(f_{ij}-f_{j}^{+}\right)}^{2}}\left({\;}_{j=1,2,\ldots ,m}^{i=1,2,\ldots ,n}\right)$$

(4) $$\text{Negative}\;\text{ideal}\;\text{solution}\;S_{i}^{-}=\sqrt{\sum_{j=1}^{m}{\left(f_{ij}-f_{j}^{-}\right)}^{2}}\left({\;}_{j=1,2,\ldots ,m}^{i=1,2,\ldots ,n}\right)$$

③ Calculate the C value of the closeness between each index and the ideal solution, and the formula is:

(5) $$C_{i}=\frac{S_{i}^{-}}{S_{i}^{-}+S_{i}^{+}}\left({\;}_{j=1,2,\ldots ,m}^{i=1,2,\ldots ,n}\right)$$

In the formula, the value range of *C*_*j*_ is [0, 1]. The larger the value, the higher the urban ecological resilience value of the research unit, and vice versa.

#### 2.3.2 Kernel density estimation

Kernel density estimation (KDE) is a nonparametric estimation method that fits a function based on the inherent characteristics of the data. This approach eliminates potential errors that could arise from manually specifying the functional form, offering distinct advantages over traditional estimation methods. The expression form is [46]:

(6) $$f\left(x\right)=\frac{1}{nh}\sum_{i=1}^{n}k\left(\frac{X_{i}-x}{h}\right)$$

where *X*_*i*_ represents the observed value; *x* is the average of the observed values; $k\left(\frac{X_{i}-x}{h}\right)$ represents a Gaussian kernel function; *n* represents the number of sample observations; and *h* represents bandwidth. This paper selects the optimal bandwidth according to the principle of minimum mean square error.

#### 2.3.3 Theil index

As a method to measure the relative difference in regional development, the Theil index can reflect the difference between and within regions and quantify their contribution to the total difference. The mathematical expression is [47]:

T=Tb+Tw

(7) $$=\sum_{1}^{k}Y_{k}\;\ln\frac{Y_{k}}{n_{k}/n}+\sum_{1}^{k}Y_{k}\left(\sum_{1}^{i}\frac{Y_{i}}{Y_{k}}\ln\frac{Y_{i}/Y_{k}}{1/n_{k}}\right)$$

where *T* represents the Theil index; *T*_*b*_ and *T*_*w*_ indicate the inter -regional and intra -regional differences, respectively, of urban ecological resilience in Guangdong Province; *k* represents the number of clusters divided by 21 prefecture level cities; and *Y*_*i*_ and *Y*_*k*_ represent the urban ecological resilience of the *i*th prefecture level city and the urban ecological resilience of group *k*, respectively. The range of the Theil index is [0, 1]. The greater the value, the greater the difference.

#### 2.3.4 Center of gravity standard deviation ellipse

Lefever [48] introduced the concept of the standard deviation ellipse in 1926. This method falls within the realm of statistical analysis for spatial data and offers the advantage of unveiling the spatial distribution and multidirectional features of the subject under investigation. In our study, we utilized four fundamental parameters—gravity center, azimuth, long axis, and short axis—to analyze the primary spatial positioning and dynamic developmental patterns of urban ecological resilience and ecological vulnerability in Guangdong Province throughout the study period. The specific calculation formula is as follows [49]:

(8) $$\bar{X}=\frac{\sum_{i=1}^{n}W_{i}X_{i}}{\sum_{i=1}^{n}W_{i}},\bar{Y}=\frac{\sum_{i=1}^{n}W_{i}Y_{i}}{\sum_{i=1}^{n}W_{i}}$$

(9) $$S=\pi \sigma_{X}\sigma_{Y}$$

where *n* is the number of cities; (*X*, *Y*) represents the barycentric coordinates of urban ecological resilience; (*X*_*i*_, *Y*_*i*_) is the geographic coordinate of each city; *W*_*i*_ represents weight; and σ_*X*_ and σ_*Y*_ represent the standard deviation along the *X* axis and *Y* axis, respectively.

#### 2.3.5 Obstacle model

A multidimensional comprehensive evaluation index for urban ecological resilience helps in assessing its multifaceted influence on the current state and future trajectory of urban ecosystems. Employing the obstacle degree model, we calculated the obstacle degree for each indicator factor and ranked them to ascertain their respective impacts on the urban ecological resilience of different cities in Guangdong Province. This quantitative analysis serves as a foundation for enhancing urban ecological resilience and advancing urban ecological preservation efforts. The calculation formula is [50]:

(10) $$F_{j}=W_{j}X_{ij}^{\prime }/W_{j}X_{ij}^{\prime }\cdot 100\%$$

where *F*_*j*_ is the obstacle degree of the jth index to urban ecological resilience; *W*_*j*_ is the index weight of item *j*; *X*_*xj*_' is the standardized value of index *j*; and *N* is the number of indicators.

#### 2.3.6 Back-propagation (BP) neural network prediction model

A three-layer BP neural network prediction model was employed to forecast urban resilience measurement values for the period from 2022 to 2030 within the MATLAB R2018b environment [51]. The model utilized The 21 urban resilience evaluation indicators outlined in Table 1 as the input layer, while the target layer represented the final output layer. The initial step involved using the input and output sample sets for training to establish the mapping relationship between the provided input and output data. Subsequently, we fed the urban resilience data for Guangdong Province spanning from 2000 to 2020 into the model to predict urban resilience levels for the years 2022–2030.

#### 2.3.7 Analysis method of the geostatistical trend

Geostatistical trend analysis plays a significant role in GIS geostatistical analysis, and it is a crucial component of this study. Utilizing the ArcGIS platform, we employed the urban resilience measurement values for Guangdong Province from 2020 to 2030 as our foundational dataset. In the trend analysis, we conducted a perspective analysis by projecting the urban resilience measurement values from 2020 to 2030 onto orthogonal planes aligned with the east-west and north-south directions. Each vertical bar within the trend analysis diagram symbolizes both the height and position of an urban resilience measurement value. By projecting these urban resilience measurements onto the orthogonal planes and drawing a best fitting line through the projection points, we aimed to simulate the changing trend of urban resilience in specific directions. This approach allows us to gain deeper insights into the future spatial development law of urban resilience [52].

## 3 Results and discussion

### 3.1 Temporal and spatial differentiation characteristics of urban ecological resilience in Guangdong Province

#### 3.1.1 Temporal evolution characteristics of urban ecological resilience in Guangdong Province

We utilized the entropy weight TOPSIS method to evaluate the urban ecological resilience of Guangdong Province spanning the years 2000–2020. This assessment yielded urban ecological resilience values for the 21 prefecture -level cities, along with the average value for the entire 21-year period, as presented in Table 2. From 2000 to 2020, the average value of urban ecological resilience in Guangdong Province was 0.366, showing an upward-trending pattern with fluctuations. The mean urban ecological resilience values of Zhuhai, Chaozhou, Qingyuan, Shenzhen, and Yangjiang significantly surpassed the provincial average (0.413, 0.410, 0.401, 0.394, and 0.387, respectively). Prior to 2015, the overall urban ecological resilience in Guangdong Province remained relatively low, with a value of 0.498. This period corresponded to the "12th Five-Year Plan," during which the province emphasized green development and the establishment of a resource-efficient and environmentally friendly society. Guangdong Province was in the initial stages of industrial restructuring and the construction of ecological civilization, leading to gradual improvements in urban ecological resilience. From 2015 to 2020, there was a notable enhancement in the overall urban ecological resilience of Guangdong Province. This suggests that within the context of the "new normal," the province's efforts in ecological civilization construction yielded remarkable results. By 2020, urban ecological resilience in Guangdong Province had increased to 0.667, marking a substantial 33.94 % increase compared to the 2015 levels. This progress aligns with the strategic imperative of vigorously establishing a wetland ecological protection system and advancing the development of a green ecological water network in the Pearl River Delta.

**Table 2:** Urban ecological resilience of Guangdong Province.

City	2000	2001	2002	2003	2004	2005	2006
Guangzhou	0.0311	0.0527	0.1891	0.0937	0.2117	0.2710	0.2320
Shaoguan	0.2244	0.2623	0.2240	0.2034	0.2117	0.2238	0.2624
Shenzhen	0.1177	0.1413	0.2110	0.1826	0.3342	0.2130	0.2891
Zhuhai	0.1361	0.1927	0.2100	0.2177	0.2727	0.2166	0.1770
Shantou	0.1278	0.0864	0.1087	0.3221	0.3448	0.2633	0.2655
Foshan	0.0497	0.0815	0.1651	0.2898	0.3085	0.1350	0.2364
Jiangmen	0.1134	0.1314	0.1522	0.2036	0.2411	0.2436	0.1993
Zhanjiang	0.3008	0.1951	0.2078	0.2334	0.3042	0.2579	0.2330
Maoming	0.1975	0.2444	0.2487	0.2072	0.2629	0.2295	0.1947
Zhaoqing	0.1812	0.1967	0.1760	0.2418	0.2332	0.1463	0.1652
Huizhou	0.1506	0.1479	0.2099	0.2759	0.2844	0.1228	0.2508
Meizhou	0.1824	0.1944	0.2178	0.2305	0.2827	0.2971	0.2510
Shanwei	0.0489	0.1016	0.1560	0.2515	0.2684	0.2384	0.3570
Heyuan	0.0883	0.1727	0.1049	0.1185	0.0875	0.1060	0.1869
Yangjiang	0.0992	0.1576	0.2214	0.1360	0.1666	0.2346	0.2493
Qingyuan	0.2027	0.2522	0.2812	0.2948	0.3359	0.2624	0.1401
Dongguan	0.1621	0.1881	0.2222	0.2143	0.3047	0.2340	0.2674
Zhongshan	0.1596	0.1815	0.2127	0.2074	0.2829	0.2197	0.2485
Chaozhou	0.1428	0.1599	0.1418	0.2127	0.3536	0.2972	0.3196
Jieyang	0.1977	0.0626	0.0909	0.1843	0.2089	0.2287	0.2530
Yunfu	0.1325	0.1179	0.1285	0.1544	0.1933	0.1499	0.1819
Average value	0.1451	0.1581	0.1848	0.2131	0.2616	0.2186	0.2362
City	2007	2008	2009	2010	2011	2012	2013
Guangzhou	0.2527	0.2538	0.2771	0.2823	0.3289	0.4171	0.5073
Shaoguan	0.2205	0.2609	0.3023	0.3242	0.3307	0.3037	0.3239
Shenzhen	0.3276	0.3533	0.3787	0.4060	0.4458	0.4448	0.4880
Zhuhai	0.1777	0.2942	0.3299	0.4129	0.4731	0.4891	0.5311
Shantou	0.2589	0.3069	0.3273	0.3481	0.3629	0.2417	0.2786
Foshan	0.2917	0.2757	0.2973	0.3037	0.3310	0.3147	0.3827
Jiangmen	0.2082	0.2291	0.2506	0.3550	0.3871	0.4085	0.4472
Zhanjiang	0.2788	0.2957	0.3251	0.3340	0.3767	0.2799	0.3133
Maoming	0.2178	0.2359	0.2119	0.1899	0.3286	0.3886	0.4224
Zhaoqing	0.2947	0.2271	0.2657	0.3918	0.4323	0.3935	0.4356
Huizhou	0.2666	0.2854	0.3265	0.2756	0.2956	0.4463	0.4724
Meizhou	0.1419	0.2970	0.3335	0.2849	0.4148	0.5568	0.3668
Shanwei	0.2704	0.2836	0.3826	0.3956	0.3807	0.4320	0.4644
Heyuan	0.2142	0.2425	0.2814	0.4209	0.3957	0.4184	0.4113
Yangjiang	0.2818	0.3221	0.3762	0.3735	0.4074	0.5071	0.4471
Qingyuan	0.1875	0.2165	0.3235	0.2981	0.3217	0.3479	0.4778
Dongguan	0.2318	0.2467	0.2569	0.2790	0.2959	0.3320	0.4390
Zhongshan	0.2075	0.2189	0.2324	0.2628	0.2813	0.3168	0.4266
Chaozhou	0.3325	0.3700	0.3997	0.4454	0.4739	0.4942	0.5182
Jieyang	0.3960	0.2588	0.276	0.2918	0.2665	0.4099	0.4163
Yunfu	0.3191	0.3074	0.3797	0.3718	0.3019	0.3219	0.3183
Average value	0.2561	0.2753	0.3112	0.3356	0.3634	0.3936	0.4233
City	2014	2015	2016	2017	2018	2019	2020
Guangzhou	0.5115	0.6043	0.6082	0.6077	0.6583	0.7096	0.6896
Shaoguan	0.3349	0.3428	0.3829	0.3804	0.4639	0.4492	0.4627
Shenzhen	0.5155	0.5501	0.5489	0.5757	0.5596	0.5591	0.6235
Zhuhai	0.5636	0.6108	0.625	0.6648	0.6822	0.7126	0.6811
Shantou	0.3110	0.3928	0.4318	0.4617	0.5034	0.5402	0.6996
Foshan	0.4332	0.4634	0.4751	0.4938	0.5997	0.6504	0.6908
Jiangmen	0.5329	0.5514	0.5644	0.5609	0.5760	0.6023	0.6287
Zhanjiang	0.4589	0.3896	0.5508	0.5418	0.6427	0.6502	0.6440
Maoming	0.4510	0.4579	0.4953	0.4503	0.5816	0.6426	0.7566
Zhaoqing	0.5120	0.5523	0.5925	0.584	0.6178	0.6580	0.7243
Huizhou	0.4962	0.5205	0.5612	0.6056	0.6411	0.6892	0.7135
Meizhou	0.4583	0.4892	0.5197	0.5495	0.5641	0.5801	0.6693
Shanwei	0.4739	0.5371	0.5272	0.5375	0.6198	0.6370	0.7328
Heyuan	0.3742	0.4185	0.4252	0.458	0.4804	0.5293	0.5511
Yangjiang	0.4886	0.5173	0.5527	0.5668	0.6203	0.6780	0.7216
Qingyuan	0.5661	0.6187	0.6316	0.6507	0.6459	0.6669	0.6901
Dongguan	0.4551	0.5206	0.634	0.6525	0.6717	0.7026	0.7315
Zhongshan	0.4467	0.5127	0.6245	0.6482	0.6696	0.6993	0.7283
Chaozhou	0.5161	0.5418	0.5636	0.542	0.5304	0.5876	0.6590
Jieyang	0.4701	0.3524	0.384	0.4435	0.4648	0.4967	0.5419
Yunfu	0.4858	0.5095	0.4883	0.5045	0.5313	0.5792	0.6701
Average value	0.4693	0.4978	0.5327	0.5466	0.5869	0.6200	0.6671

**Fig. 2:**
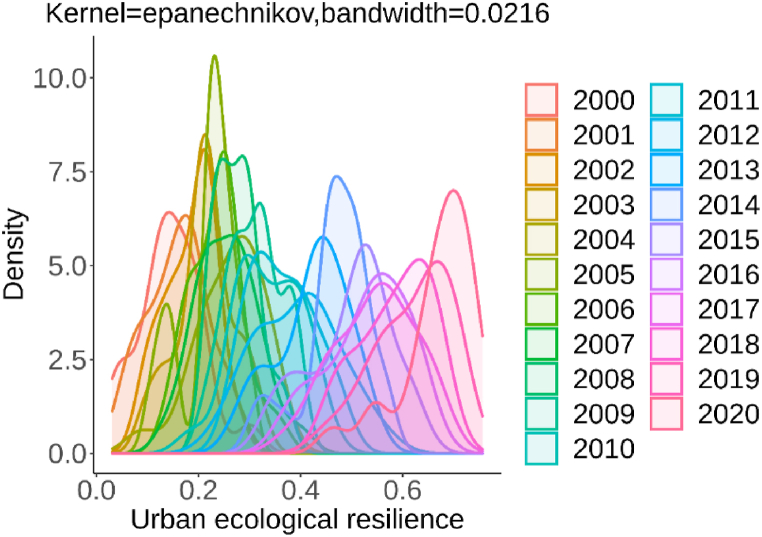
Kernel density distribution of urban ecological resilience in Guangdong Province.

To gain deeper insights into the temporal dynamics of urban ecological resilience in Guangdong Province, we conducted an estimation of its kernel density for the years 2000, 2005, 2010, 2015, and 2020, as depicted in Fig. 2. Over this two-decade span, there was a noticeable rightward shift in the position of the kernel density curve, signaling the sustained growth of urban ecological resilience within the Province. The curve's shape transformed from a sharp peak to a broader one, with a continuous decrease in peak height. This phenomenon suggested a diminishing gap in urban ecological resilience among the cities in Guangdong Province. Additionally, the kernel density curve exhibited a distinctive bimodal distribution, with the primary peak significantly outweighing the secondary peak. This indicated a substantial polarization in the urban ecological resilience of Guangdong Province, reflecting marked spatial imbalances. Furthermore, throughout the years from 2000 to 2020, the right tail of the kernel density curve consistently persisted, with a widening trend observed in 2020. This suggests that the majority of prefecture-level cities concentrated their ecological resilience at lower values, with only a few cities approaching higher values. This evolving trend can be characterized as "low concentration, high convergence."

#### 3.1.2 Spatial evolution characteristics of urban ecological resilience in Guangdong Province

**Fig. 3:**
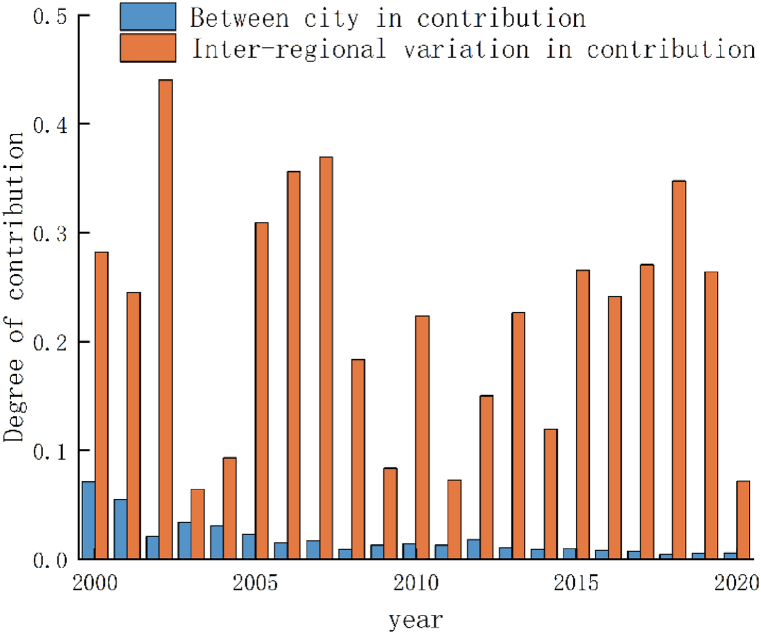
Theil index contribution.

**Fig. 4:**
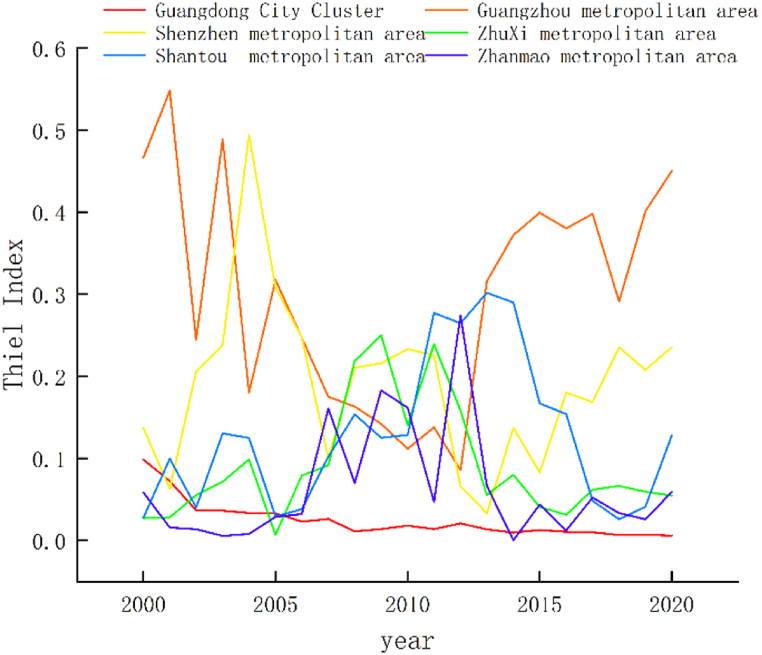
Theil index.

Based on the preceding analysis, a notable spatial imbalance has been identified in the urban ecological resilience of Guangdong Province. Consequently, in accordance with the territorial and spatial planning coordination guidelines for the Guangdong metropolitan area issued by the Department of Natural Resources of Guangdong Province in 2022, Guangdong Province has been delineated into five metropolitan areas: Guangzhou metropolitan area (comprising Guangzhou, Foshan, Zhaoqing, and Qingyuan); Shenzhen metropolitan area (encompassing Shenzhen, Dongguan, and Huizhou); Zhuxi metropolitan area (including Zhuhai, Zhongshan, and Jiangmen); Shantou metropolitan area (comprising Shantou, Chaozhou, and Jieyang); and Zhangmao metropolitan area (encompassing Zhanjiang and Maoming). These were analyzed for spatial differences using the Theil index (Fig. 3, Fig. 4).

Between 2000 and 2020, the Theil index in Guangdong Province exhibited fluctuations within the range of 0.05–0.10. During this period, Guangdong Province emphasized its commitment to green leadership and the advancement of ecological civilization construction. Cities at all administrative levels intensified their environmental oversight and remediation efforts, leading to continuous enhancements in the quality of human settlements throughout the region. Additionally, the implementation of a coordinated regional development strategy contributed to a discernible downward trajectory in the Theil index.

When considering the regional perspective, it becomes evident that the contribution of inter-regional disparities in urban ecological resilience within Guangdong Province consistently outweighed that of intra-regional disparities, thus forming a distinct "W"-shaped pattern. This pattern suggests that the primary source of regional divergence in urban ecological resilience in Guangdong Province stemmed from inter-regional disparities, and the imbalance among the five regions served as a pivotal constraint on the province's ecological civilization construction and high-quality development. The Theil index of the metropolitan areas was highest to lowest in the order of Guangzhou metropolitan area, Shenzhen metropolitan area, Shantou metropolitan area, Zhuxi metropolitan area, and Zhanmao metropolitan area, showing that the difference in urban ecological resilience of Guangzhou Metropolitan area is the largest, while that of Zhanmao Metropolitan area was the lowest.

**Fig. 5:**
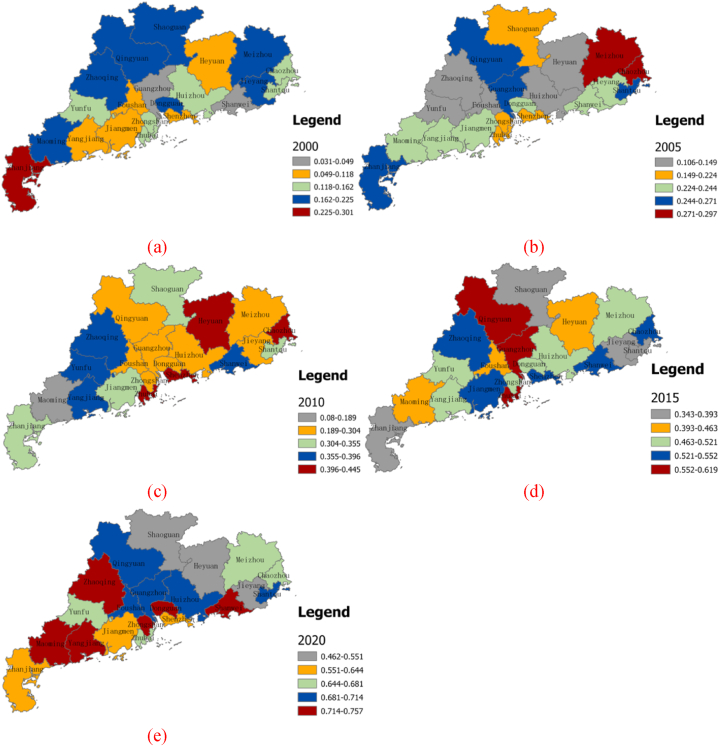
Spatial distribution of urban ecological resilience of Guangdong Province in 2000, 2005, 2010, 2015 and 2020.

We used ArcGIS10.5 software to visualize the spatial evolution of urban ecological resilience in Guangdong Province. Employing the Jenks natural discontinuity classification method, we categorized the urban ecological resilience of the 21 prefecture-level cities in Guangdong Province into five types: high value, high-median value, median value, low value, and low-median value areas, as illustrated in Fig. 5. In broad terms, the urban ecological resilience of Guangdong Province exhibited a pattern characterized by the clustering of low-value areas and the dispersion of high-value areas. In the year 2000, the range of urban ecological resilience values in Guangdong Province spanned from 0.010 to 0.301. High, median, and low-value areas collectively dominated the urban ecological resilience landscape, encompassing 85.71 % of the total area. High-value areas comprised just one prefecture-level city. As we progressed to 2005, urban ecological resilience became increasingly dominated by middle, low, and low-median value areas, accounting for 71.43 % of the overall area. Notably, Huizhou and Yunfu shifted from the median to the low-value category, while Foshan and Heyuan transitioned from low to very low-value areas.

**Fig. 6:**
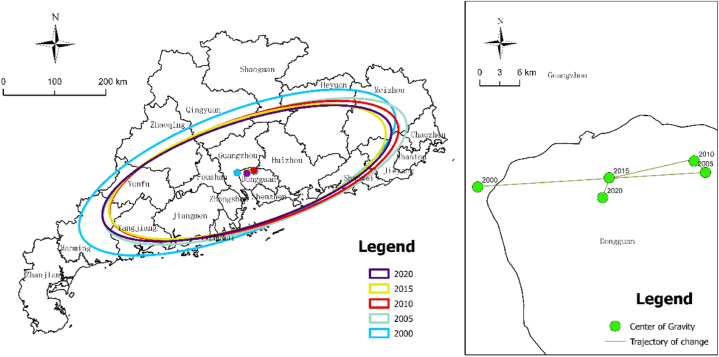
Standard deviation ellipse and gravity center trajectory of urban ecological resilience in Guangdong Province.

This phenomenon may be attributed to the initial phase of ecological civilization construction, spanning from 2000 to 2005, during which various regions sought immediate effects through administrative measures, often lacking long-term sustainability. In 2010, Guangdong Province significantly bolstered its efforts in controlling environmental pollution and ecological protection. This led to a decrease in the number of cities categorized as having low and very low-value urban ecological resilience, while the number of cities in the high-value areas increased to four. By 2015, urban ecological resilience had shown noticeable improvement. Areas with Very high, high, and median ecological resilience values collectively accounted for 61.90 % of the total area. Meanwhile, the number of prefecture-level cities in the low-value category decreased to three. In 2020, the range of urban ecological resilience values in Guangdong Province spanned from 0.189 to 0.445. High, high-median, and medium-value areas of urban ecological resilience increased to 71.43 %, while the number of low-value areas and prefecture-level cities remained at three. This marked a significant overall enhancement in urban ecological resilience compared to the 2015 levels. In summary, the progression of urban ecological resilience in Guangdong Province exhibits a pattern of oscillation and upward spiral evolution. Specifically, the spatial distribution of urban ecological resilience in Guangdong Province was characterized by high values in the Shenzhen metropolitan area, low values in the Zhanmao metropolitan area, and polarization in the Guangzhou metropolitan area. These observations align with the analysis results derived from the Theil index. Furthermore, to gain deeper insights into the spatial dynamics of urban ecological resilience in Guangdong Province, we employed the center of gravity standard deviation ellipse to construct a spatial dynamic evolution map from 2000 to 2020 (Fig. 6). Over this period, the standard deviation ellipse representing urban ecological resilience in Guangdong Province displayed a northeast-to-southwest pattern and shifted predominantly northeastward. Its total coverage area decreased by 2216.06 km^2^. Additionally, the azimuth angle exhibited fluctuations, decreasing from 68.70° in 2000 to 71.91° in 2010, then subsequently decreasing to 71.90° in 2020, indicating a clockwise shift in urban ecological resilience in Guangdong Province. The main axis of the standard deviation ellipse shortened from 295.00 km in 2000 to 265.31 km in 2020, signifying a convergence of urban ecological resilience along the northeast-to-southwest axis. Conversely, the secondary axis of the standard deviation ellipse exhibited fluctuations, suggesting temporary instability in the north-south spatial evolution of urban ecological resilience in Guangdong Province.

### 3.2 Identification of barriers to urban ecological resilience in Guangdong Province

**Fig. 7:**
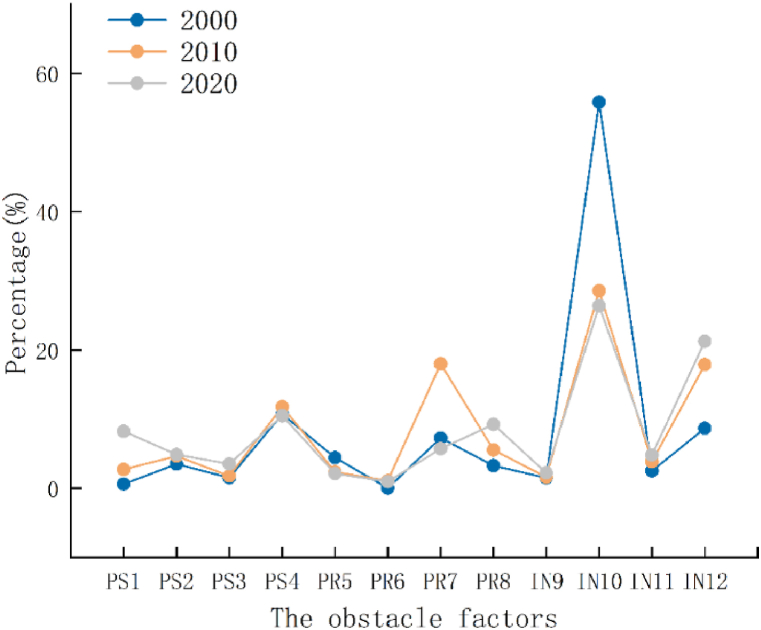
The obstacle factors of urban ecological resilience of Guangdong in 2000, 2010, and 2020.

The barrier degree model was used to analyze the barriers to urban ecological resilience in Guangdong Province, determine the resistance factors affecting urban ecological resilience, screen out the top three significant barrier factors, and explore the internal source of urban ecological resilience improvement in Guangdong Province. Considering the large number of data samples in the 10 years from 2000 to 2020, the data for 2000, 2010, and 2020 were selected as samples for the barrier factor analysis (Fig. 7, Table 3).

**Table 3:** Top three obstacle factors of urban ecological resilience in Guangdong Province (%).

City	2000			2010			2020		
	1	2	3	1	2	3	1	2	3
Guangzhou	RS1 (46.91)	RS3 (21.23)	RP7 (19.96)	IN12(23.19)	IN9(18.36)	RS1 (16.13)	IN12(21.14)	IN10(19.05)	IN10(11.63)
Shaoguan	IN12(48.07)	RS2 (38.71)	RP8 (12.58)	RP6 (39.42)	IN10(20.72)	RP7 (12.64)	RP6 (27.62)	IN10(21.83)	RP7 (16.61)
Shenzhen	RP7 (70.05)	RS2 (18.01)	RS3 (7.07)	RP7 (24.04)	IN12(22.86)	RP8 (15.34)	IN12(29.45)	RP7 (20.49)	IN11(16.19)
Zhuhai	RS1 (45.92)	RP6 (36.42)	RS2 (11.69)	IN11(28.48)	RP6 (27.01)	RP3 (13.58)	IN12(26.96)	RP7 (13.83)	RS3 (13.46)
Shantou	IN11(36.35)	RS2 (20.36)	RP8 (18.58)	RP7 (36.67)	RS4 (19.75)	RP8 (11.79)	IN12(26.25)	RP4 (18.09)	RS4 (13.11)
Foshan	RS1 (42.42)	RS2 (35.21)	RS4 (17.39)	RP6 (33.25)	RS3 (19.49)	RS1 (11.77)	IN11(26.58)	RP6 (18.51)	IN10(14.62)
Jiangmen	RS1 (72.42)	RS2 (12.23）	RP8 (9.93)	RP6 (35.99)	RS3 (18.14)	IN11(16.17)	IN11(29.21)	RS7 (20.33)	RP8 (14.98)
Zhanjiang	RP6 (33.41)	RP7 (31.31)	IN10(19.66)	RP6 (36.32)	IN11(20.92)	RS3 (14.03)	IN12(23.51)	RP6 (19.57)	IN10(15.68)
Maoming	RP6 (54.31)	RS1 (32.89)	RP7 (4.08)	RS3 (29.97)	IN12(22.04)	RS2 (13.08)	IN11(24.27)	RP6 (16.89)	RS1 (13.01)
Zhaoqing	RS1 (53.91)	RP6 (24.56)	RP7 (11.56)	RP6 (31.91)	RS4 (12.81)	RS2 (11.52)	IN12(25.25)	RP7 (17.65)	RP8 (13.01)
Huizhou	RP6 (71.15)	RS2 (16.89)	RS3 (6.59)	RP6 (21.21)	RS4 (18.76)	IN10(17.21)	IN12(25.73)	RP7 (17.91)	IN10(14.16)
Meizhou	RS2 (34.51)	RP6 (17.89)	IN12(17.61)	RS3 (28.01)	RP3 (26.81)	IN11(23.91)	IN12(43.31)	RP6 (31.27)	IN12(23.18)
Shanwei	IN11(51.62)	RP8 (25.19)	RS3 (12.35)	RP7 (24.38)	IN11(19.15)	RS4 (15.38)	IN12(25.06)	RP7 (17.44)	RS2 (13.43)
Heyuan	RP7 (47.01)	IN11(23.22)	RP8 (21.50)	RP7 (32.30)	IN12(22.58)	IN11(21.95)	IN12(28.09)	RP7 (19.34)	IN11(15.46)
Yangjiang	RS2 (42.62)	RP7 (23.06)	RP8 (14.20)	RP7 (34.21)	IN12(19.71)	IN11(13.33)	IN12(22.04)	RP7 (17.71)	IN11(14.00)
Qingyuan	RP7 (51.89)	RS2 (23.75)	IN11(17.89)	IN12(22.94)	RS2 (19.09)	IN11(18.24)	RP7 (18.52)	IN12(18.03)	IN11(14.63)
Dongguan	RP7 (78.84)	RS3 (9.38)	RP5 (6.71)	RS4 (28.36)	RS2 (19.99)	RP8 (17.62)	IN12(25.10)	RP7 (17.47)	IN1(13.81)
Zhongshan	RP7 (80.07)	RS3 (9.52)	RP5 (5.71)	RS4 (25.87)	RS2 (19.24)	RP8 (18.70)	IN12(25.21)	RP7 (17.55)	IN11(13.86)
Chaozhou	RS2 (66.56)	RP8 (23.55)	RP5 (5.23)	RP7 (28.69)	IN12(25.10)	IN11(18.14)	IN12(27.86)	RP7 (19.39)	RP8 (14.29)
Jieyang	IN11(51.09)	RP8 (27.93)	RS2 (10.96)	RP8 (20.90)	RS4 (20.11)	IN11(15.38)	RP7 (23.28)	RP8 (17.38)	RS2 (17.32)
Yunfu	RS2 (58.18)	IN11(22.55)	RS3 (8.73)	RP7 (29.33)	IN11(18.75)	IN12(17.98)	IN12(27.40)	RP7 (19.07)	RP8 (14.06)

Throughout the study period, the change in barrier factors in Guangdong Province exhibited a consistent pattern with the spatial evolution of urban ecological resilience. In the year 2000, Guangdong was still in the early stages of ecological civilization construction, and Urban ecological resilience was primarily concentrated in areas with moderate to lower values. The primary barrier factors at this time were the number of patent grants, the biological abundance index, and the number of college students. These three factors had barrier degrees of 55.84 %, 10.72 %, and 8.67 %, respectively. By the year 2010, the internal factors impeding the urban ecological resilience of Guangdong Province began to shift from resistance within the ecosystem to renewal. The prominent obstacles were the number of patents granted, the harmless disposal rate of domestic waste, and the number of college students. The combined effect of these three factors accounted for 64.45 %. This shift may be attributed to the post-2008 economic crisis period, during which Guangdong's economy required a swift recovery, leading to rapid urban land expansion. The rapid urbanization placed significant pressure on the urban ecological environment. Additionally, China's gradual relaxation of the two-child policy for urban populations increased population density, gradually reducing the carrying capacity of the urban ecological environment. In the context of the 13th Five -Year Plan, the urban ecological infrastructure in Guangdong Province was notably enhanced. By 2020, the key internal factor affecting urban ecological resilience was responsiveness, specifically encompassing the number of patents granted, the number of college students, and the biological abundance index. These factors had obstacle degrees of 26.41 %, 21.27 %, and 10.48 %, respectively. Amidst the ongoing urbanization and industrialization processes, it is imperative to reinforce the construction of infrastructure for urban emission reduction and pollution control while simultaneously enhancing the responsiveness of urban ecosystems.

From the perspective of cities at all levels (Table 3), during the study period, the per capita water resources were one of the main obstacles to the ecological resilience of Guangzhou, with the highest obstacle degree of 46.91 %. As the capital city of Guangdong Province, the scale of urban construction and population concentration is unprecedented. While the regional economy is developing positively, it presents great challenges to ecosystem resilience. However, in addition to the per capita green space area of parks, normalized vegetation index, and proportion of environmental protection in financial expenditure, Shenzhen's urban ecological resilience is restricted by the harmless disposal rate of domestic waste, with the highest obstacle degree of 70.05 %. Therefore, the above two prefecture-level cities should also strengthen the construction and maintenance of the urban ecological background, improve the resistance of urban ecosystems, and build ecological security barriers, while focusing on the improvement of urban ecosystem responsiveness.

### 3.3 Dynamic simulation analysis of urban resilience in Guangdong Province

#### 3.3.1 Prediction of urban resilience level evolution trend

**Fig. 8:**
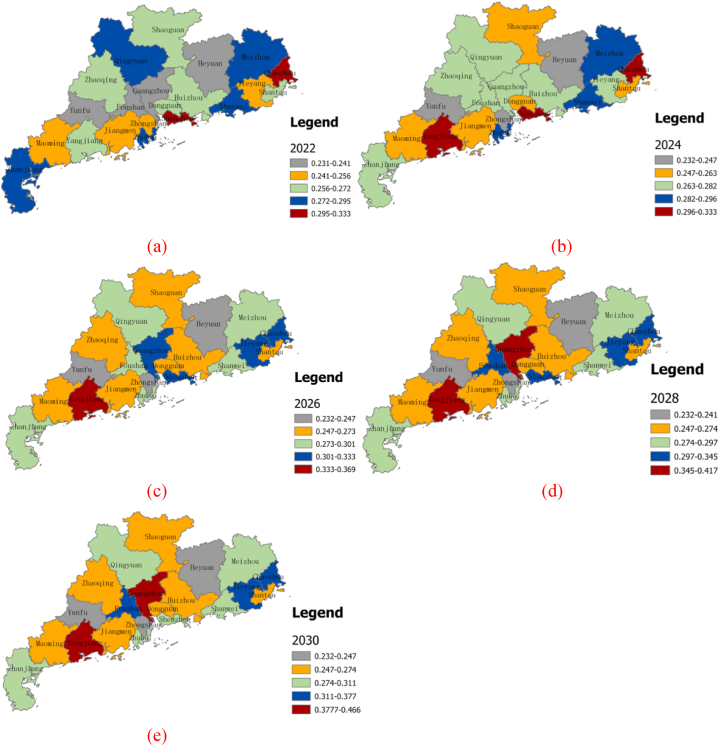
Evolution trend of the urban resilience level of Guandong province in 2022–2030.

The BP neural network model was employed to predict the changing trend of urban resilience in Guangdong Province from 2022 to 2030, as illustrated in Fig. 8. The prediction results indicated an overall slow development of urban resilience in Guangdong Province, with the degree of resilience gradually decreasing. The areas characterized by low and median resilience values predominated, while cities with higher levels of resilience constituted a smaller proportion. Within this context, certain cities exhibited distinct patterns. Heyuan and Yunfu displayed a low level of resilience, and their trends were relatively stable, primarily falling within the low-value resilience areas. Qingyuan, Meizhou, Zhanjiang, and Shanwei showed a transitioning trend from high to medium resilience levels, whereas Zhaoqing, Shaoguan, and Huizhou demonstrated a trend of transitioning from medium to low resilience levels. In contrast, Shenzhen and Chaozhou maintained a high level of urban resilience, albeit with fluctuating transitions between high-value resilience areas. Yangjiang exhibited the highest level of resilience and maintained a relatively stable trend. It was predominantly characterized by areas with high resilience values, though areas with very high resilience values also constituted a significant portion.

#### 3.3.2 Prediction of spatial development trendline of urban resilience level

**Fig. 9:**
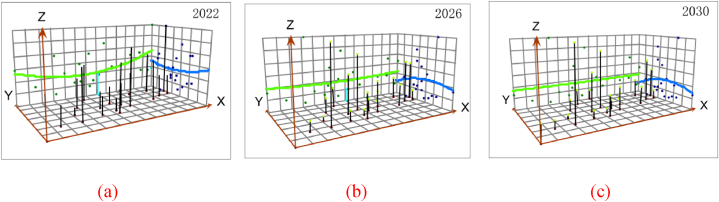
Urban ecological resilience of Guangdong Province in 2022, 2026, and 2030.

The trendline prediction chart for the urban resilience levels in Guangdong Province in 2022, 2026, and 2030 (Fig. 9) provides insights into the distribution characteristics and spatial development trends of urban resilience from 2022 to 2030. In the chart, the green line represents the spatial trend in the east-west direction, while the blue line represents the trend in the north-south direction. The analysis reveals that, in general, the urban resilience levels across Guangdong Province exhibit noticeable spatial disparities in both the east-west and north-south directions. During the period from 2022 to 2030, urban resilience levels are expected to gradually increase. in the east-west direction, the urban resilience level follows a “U”-shaped development trend, with relatively subtle changes anticipated from 2022 to 2030. Conversely, in the north-south direction, the trend indicates higher resilience levels in the south and lower levels in the north.

## 4 Conclusion and suggestions

This study builds upon previous research results [35, 43],utilizing the resilience assessment framework, which encompasses the dimensions of resistance, response, and renewal, this study delved into the measurement, spatial and temporal variations, hindering factors, and evolutionary patterns of urban ecological resilience in Guangdong Province. The findings can be summarized as follows.

(1) Since the year 2000, the average urban ecological resilience score for Guangdong Province has maintained a value of 0.366, displaying a fluctuating upward trend. Specifically, the period between 2000 and 2005 witnessed a generally low urban ecological resilience level in the province, which improved notably after 2010. Furthermore, a clear polarization in ecological resilience emerged among different regions, forming a trend characterized by low concentration and high convergence.
(2) The spatial distribution of urban ecological resilience across Guangdong Province exhibited notable disparities, marked by clusters of low resilience values and dispersion of high resilience values, predominantly concentrated in areas with median to high values. For instance, the Shenzhen metropolitan area exhibited high Urban ecological resilience, while the Zhanmao metropolitan area displayed lower resilience levels. In contrast, the Guangzhou Metropolitan Area showed significant polarization in resilience. These regional disparities primarily stemmed from inter-regional distinctions, with an overall trend moving from the northeast to the southwest and gradually shifting towards the northeast, resulting in a reduction in the total coverage area by 2216.06 km^2^.
(3) Over the study duration, the primary impediments to urban ecological resilience in Guangdong Province were associated with factors such as the rate of safe domestic waste disposal, the number of college students per 10,000 inhabitants, the number of R&D personnel per 10,000 labor force participants, and the per capita park green space area. This highlights that the ability of urban ecosystems to cope with disruptions and the capacity for learning and renewal within these ecosystems represent weak links in enhancing the urban ecological resilience of cities at all levels. Notably, the Per capita water resources posed a significant obstacle to ecological resilience in Guangzhou, with the highest obstacle rating of 46.91 %. In addition to the per capita green space area in parks, factors such as the normalized vegetation index and the proportion of environmental protection in financial expenditure also hindered Shenzhen's urban ecological resilience. Among these factors, the rate of safe domestic waste disposal held the highest obstacle factor at 70.05 %.
(4) Predictions regarding the evolution of urban resilience grades in Guangdong Province from 2022 to 2030 indicate a slow, gradual decline in the overall urban resilience of the province. The areas with low and medium resilience values will remain predominant, with only a limited number of cities falling into the high resilience category. Spatially, the east-west and north-south disparities in urban resilience will remain pronounced. The change trend from 2022 to 2030 appears to be less discernible, with a higher level of resilience in the south and a lower level in the north in the south-north direction.

The Pearl River stands as a formidable waterway, ranking as China's third-longest river and second -largest in terms of water flow. Encompassing the provinces of Yunnan, Guizhou, Guangxi, Guangdong, Hunan, and Jiangxi, the Pearl River Basin sprawls across approximately 450,000 square kilometers, sheltering a population of around 124 million. Recognizing its pivotal role in the overall national development, it becomes imperative to prioritize The ecological preservation and high-quality development of this vast region. It is important to acknowledge that this study solely focused on assessing the urban ecological resilience within Guangdong Province. Consequently, it is challenging to present a comprehensive picture of the urban ecological resilience across the entire Pearl River Valley urban agglomeration. In forthcoming research endeavors, our aim is to conduct a comprehensive evaluation of the urban ecological resilience in the upper, middle, and lower reaches of the Pearl River Valley. This expanded scope will enable a more profound exploration of its spatial interconnections.

The empirical findings of this study underscore several key observations. Firstly, it highlights the overarching low level of urban ecological resilience across Guangdong Province, magnifying the significance of spatial imbalances and regional disparities. The principal hindrances to bolstering urban ecological resilience predominantly hinge on the responsiveness and renewal capacity of urban ecosystems.

In light of these findings, we put forth the following recommendations.

(1) Establishment of an ecological civilization tailored to the distinct needs of various regions, with a primary focus on promoting sustainable urban ecological resilience. Special attention should be directed towards designating the Zhuxi metropolitan area as a highly efficient ecological, economic zone and reinforcing the protection and restoration of natural wetlands in the Pearl River Delta. Additionally, encouraging the transformation and upgradation of key marine industries, alongside stringent adherence to the ecological protection red line, is vital. Addressing the issues of High energy consumption and pollution in the Zhanmao metropolitan area is also imperative, with an emphasis on creating an ecological industrial chain through regulatory and structural adjustments.
(2) Strengthening the construction of urban green infrastructure and enhancing the responsiveness of urban ecosystems. This can be achieved by expanding urban green spaces, increasing green coverage, and strategically designing urban green infrastructure to fulfill multifaceted functions that augment urban ecological carrying capacity. Investments in pollution control and emission reduction facilities should be ramped up while the operational efficiency of green infrastructure is improved.
(3) Cultivating new strengths for renewal and development while augmenting the renewal capabilities of urban ecosystems. Emphasis should be placed on scientific experimentation and technological breakthroughs in areas such as water security, ecological protection, and vegetation restoration. Furthermore, the region can be revitalized through the application of science and technology, with efforts aimed at expanding the pool of highly skilled ecological governance professionals. Additionally, promoting high-quality discipline development and intensive talent training in higher education institutions can be instrumental. Establishing a regional renewal platform and talent-sharing mechanism, along with facilitating the integration of science and education, will be essential. accelerating the capitalization and industrialization of scientific and technological achievements in the realm of future renewals should also be pursued.

At the same time, it is pertinent to acknowledge the limitations of this study. Firstly, in constructing the assessment system, ecological resilience primarily considers the impact of human activities on the ecological environment, with limited consideration of natural factors such as land type, climate fluctuations, and biodiversity. Future research should aim to incorporate a broader range of natural environment-related variables to develop a more comprehensive evaluation system that accounts for coordinated development. Secondly, the research period was confined to the years 2000–2020 due to data availability constraints and standardization issues. In the future, with updated data, we aspire to delve deeper into the evolving trends and inherent patterns of urban ecological resilience and efficiency in Guangdong Province in terms of high-quality development.

## Funding

This study received support from the following sources: a grant from the Guangzhou Huashang College (No.2022HSKT02); a grant from the Guangzhou Huashang College (No.2021HSXK10); the Philosophy and Social Sciences of Guangzhou in the 14th Five-year Period (2023GZGJ314).

## Data availability

Data will be made available on request.

## CRediT authorship contribution statement

**Zhenjie Liao:** Writing – review & editing, Writing – original draft, Software, Formal analysis, Conceptualization. **Lijuan Zhang:** Writing – review & editing, Writing – original draft, Data curation.

## Declaration of competing interest

The authors declare no competing interests.
